# Recent Developments in Data Independent Acquisition (DIA) Mass Spectrometry: Application of Quantitative Analysis of the Brain Proteome

**DOI:** 10.3389/fnmol.2020.564446

**Published:** 2020-12-23

**Authors:** Ka Wan Li, Miguel A. Gonzalez-Lozano, Frank Koopmans, August B. Smit

**Affiliations:** Department of Molecular and Cellular Neurobiology, Center for Neurogenomics and Cognitive Research, Amsterdam Neuroscience, Faculty of Science, Vrije Universiteit Amsterdam, Amsterdam, Netherlands

**Keywords:** proteomics, neuroscience, brain, synapse, LC-MS, quantitative analyses

## Abstract

Mass spectrometry is the driving force behind current brain proteome analysis. In a typical proteomics approach, a protein isolate is digested into tryptic peptides and then analyzed by liquid chromatography–mass spectrometry. The recent advancements in data independent acquisition (DIA) mass spectrometry provide higher sensitivity and protein coverage than the classic data dependent acquisition. DIA cycles through a pre-defined set of peptide precursor isolation windows stepping through 400–1,200 m/z across the whole liquid chromatography gradient. All peptides within an isolation window are fragmented simultaneously and detected by tandem mass spectrometry. Peptides are identified by matching the ion peaks in a mass spectrum to a spectral library that contains information of the peptide fragment ions' pattern and its chromatography elution time. Currently, there are several reports on DIA in brain research, in particular the quantitative analysis of cellular and synaptic proteomes to reveal the spatial and/or temporal changes of proteins that underlie neuronal plasticity and disease mechanisms. Protocols in DIA are continuously improving in both acquisition and data analysis. The depth of analysis is currently approaching proteome-wide coverage, while maintaining high reproducibility in a stable and standardisable MS environment. DIA can be positioned as the method of choice for routine proteome analysis in basic brain research and clinical applications.

## Introduction

The brain is the most complex organ in the human body. It consists of about 85 billion neurons of different types, and an equal number of non-neuronal cells (Herculano-Houzel, 2009). The neurons communicate within the central nervous system and with the body periphery through the use of hundreds of trillions of synapses for neurotransmission. In support of these, glia cells nourish the brain and in part regulate neurotransmission (Allen, 2014). The brain's computational capacity to act as signal receiver, integrator, and output device crucially depends on its neuronal network connectivity and synaptic features (Caroni et al., 2012). In particular, activity-dependent plasticity of synapses in specific brain regions is thought to underlie learning and memory (von Engelhardt et al., 2010; Humeau and Choquet, 2019), whereas aberrant spatial-temporal changes of cells and synapses/organelles are underlying causes of psychiatric and neurodegenerative disorders (Counotte et al., 2011; Kanellopoulos et al., 2020).

Over the last decade, advances in mass spectrometry-based proteomics have contributed significantly to the understanding of the underlying molecular mechanisms at stake in health and disease. In particular, proteomics has been extensively used to catalog protein constituents, specifically the synapse. More recently, quantitative proteomics that interrogates thousands of proteins has been applied to examine specific regions of the brain (Sharma et al., 2015), synaptic proteomes (Biesemann et al., 2014; Loh et al., 2016; Pandya et al., 2017), neurodegenerative brain tissues (Hondius et al., 2016; Li et al., 2019; Bai et al., 2020; Johnson et al., 2020), and cell cultures (Frese et al., 2017). Although data dependent acquisition (DDA) has been the most commonly used comprehensive and quantitative proteomics methodology, more recently, data independent acquisition (DIA) has gained popularity due to its improved detection and quantitation. Multiplexed proteomics, based on isobaric mass tags such as the commercially available iTRAQ and TMT reagents, is another popular quantitative proteomics technology that allows the simultaneous identification and quantitation of up to 16 samples (using TMTpro 16-plex labeling reagents). This method is outside the scope of the present review; we refer the reader to another recent review (Pappireddi et al., 2019).

## Quantitative Proteomics by Data Dependent Acquisition (DDA)

Proteomics critically depends on the use of a mass spectrometer (MS) with good mass accuracy and high sensitivity. In a typical proteomics approach, proteins biochemically extracted from the tissue or organelle of interest are enzymatically digested into tryptic peptides. Peptides are fractionated by liquid chromatography (LC), usually based on their hydrophobicity on a reversed phase column. The eluted peptides are electro-sprayed online into the MS for analysis.

DDA has been the predominant method for quantitative proteomics. The eluted peptides from LC are detected by first stage MS1 generally within a mass range 400–1,200 m/z. A fixed number of most abundant peptides are then selected sequentially for second stage tandem mass spectrometry (MS/MS). Depending on the scan speed and sensitivity of the MS, 3–20 MS/MS can be performed in each cycle. Once the high intensity peak has been sequenced it will be excluded for re-analysis so that less abundant peptides can be identified. MS/MS of each selected precursor generates a fragment ion spectrum, which can be compared to all predicted fragments for all hypothetical peptides of the appropriate molecular mass. The most common format for this protein sequence database is the FASTA format. Each peptide match can then be linked to its corresponding protein.

In case there are more co-eluted peptides than the maximum number of MS/MS performed, the less abundant ones would not be selected for MS/MS and escape identification (Michalski et al., 2011). Furthermore, due to the semi-stochastic nature of precursor selection for MS/MS, each peptide may not be consistently detected in all samples, resulting in many missing values.

A single cycle of MS1 and MS/MS analysis usually takes 2–3 s, but it can vary depending on the LC-MS configuration. The MS switches back to the MS1 to detect and quantify the peptides and repeat the sequential MS/MS analysis. For accurate quantitation, enough data points should be acquired to accurately observe each peptide's elution profile. The total peptide peak area under the MS1 elution profile represents the quantity of the peptide. An alternative method is spectral counting where quantification of a protein relies on the number of spectra identified. The run time per LC-MS/MS is often between 0.5 and 2 h, but longer run times have also been reported that could increase the number of identified proteins (Muntel et al., 2019).

## Data Independent Acquisition (DIA) is Emerging as Method of Choice for Quantitative Proteomics

DIA (Ludwig et al., 2018; Zhang et al., 2020), also known as SWATH (Liu et al., 2013), enables the quantitation of thousands of proteins with low variation and high reproducibility, as demonstrated in a multi-laboratory evaluation study (Collins et al., 2017). In principle, DIA interrogates all peptides within the selected m/z windows, typically between 400 and 1,200 m/z, that contain >90% of all tryptic peptides (Koopmans et al., 2018a; Pino et al., 2020). First, the MS1 scans the entire mass range in one go. In the MS/MS mode, the precursor isolation window of 25 m/z is initially set at, for example, 400–425 m/z. All the peptides within this mass range would be fragmented and detected simultaneously for 50–100 ms. Then, the isolation window steps up by another 25 m/z to 425–450 m/z for peptide fragmentation and detection. This process repeats 32 times, stepping through the whole mass range. A single cycle of MS1 and MS/MS usually takes 3 s, and cycles through the whole LC gradient. As MS/MS fragments, all the peptide precursors within a mass range of interest are used, and a highly complex fragment ion mass spectrum is generated. This is challenging for data analysis using a conventional genome-wide species-specific database. While proteins from DIA data can be identified by using a search engine, such as DIA-Umpire (Tsou et al., 2015), PECAN (Ting et al., 2017), or DirectDIA (Koopmans et al., 2018a; Muntel et al., 2019) in Spectronaut, a higher coverage of protein identities is achieved when the search is performed with a project-specific spectral library (Koopmans et al., 2018a). A project-specific spectral library is typically acquired from multiple fractionated DDA analysis of the same type of sample on the same instrument, and searched against a protein sequence database to identify peptides. The library is comprised of the elution time of each identified peptide and its fragment ions' pattern. Matching of the elution time and fragment ions' pattern from the DIA data to the spectral library aids with peptide detection. DIA MS/MS quantification is reported as the sum of the integrated fragment ion peak areas.

For a complex sample, the 25 m/z window may contain too many peptides that could create an interference problem, i.e., some fragment ions from different co-eluted precursors may have very similar masses and be difficult to distinguish. Furthermore, the fragment ion intensities of the lower abundant peptides may be suppressed. For this reason, current DIA protocols often use isolation windows <10 m/z. However, this narrower isolation window requires more MS/MS steps per cycle to cover the same overall m/z range, which increases the cycle time and therefore reduces the number of measurement points per peptide. This could compromise quantitation. An alternative is to use variable isolation windows. For example, a narrower window is set for mass ranges within 400–800 m/z because the majority of peptides are contained in this mass range, and a wider window for >800 m/z that has a lower peptide complexity.

**Figure 1:**
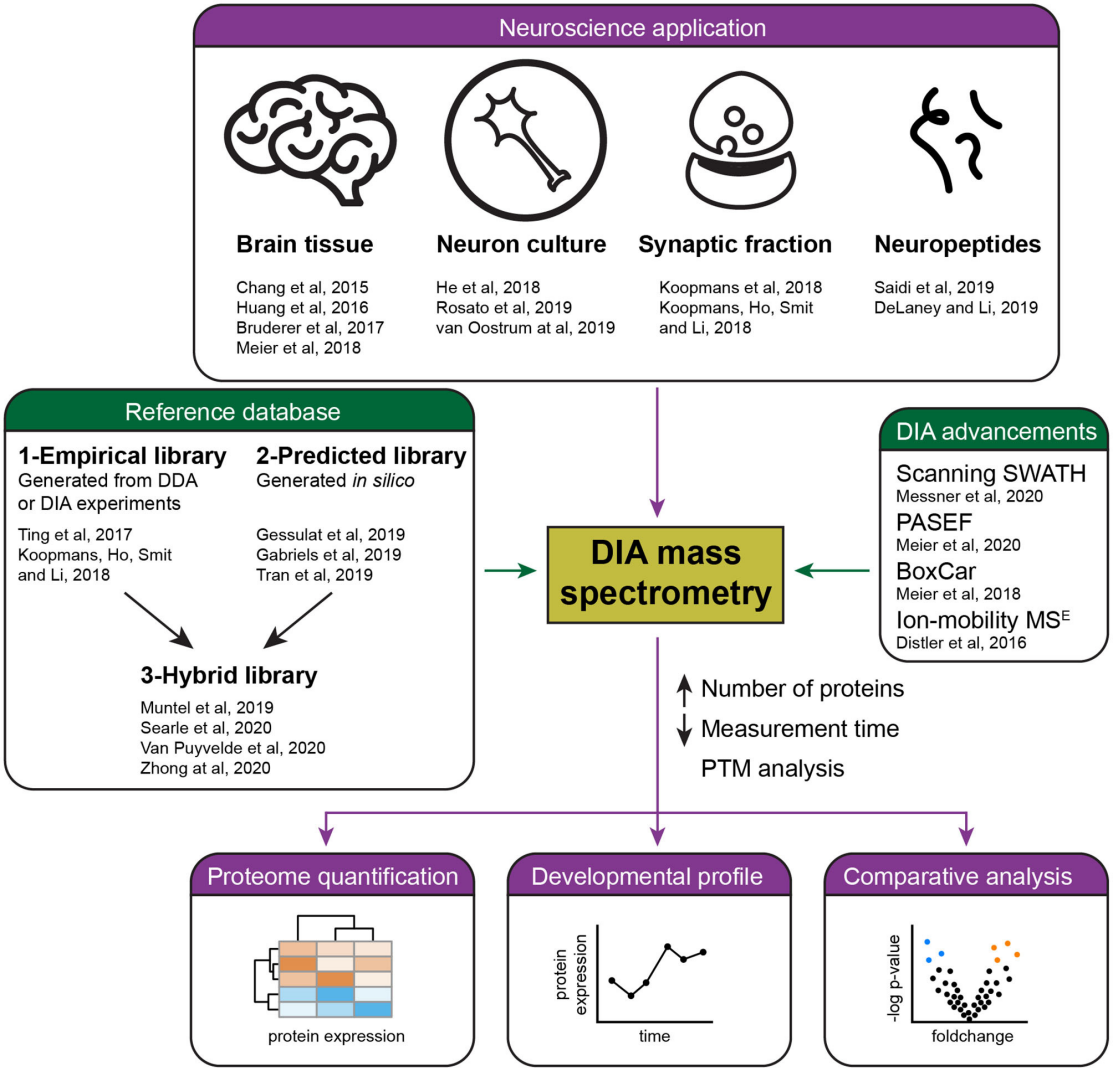
Summary of current applications and advances of DIA mass spectrometry in neuroproteomics. Several studies (purple) have successfully applied DIA mass spectrometry for the quantification of the brain proteome, including the analysis of developmental and pathological protein changes. Recent advances in DIA (green) have allowed the reliable quantification of a higher number of proteins in less time by the development of acquisition methods and improved references databases, either empirical, predicted, or hybrid libraries.

DIA is finding various applications in brain research (Figure 1). An early study examined the synaptic proteome in Alzheimer's disease patients (Chang et al., 2015), however, it still had to rely on the use of a sub-optimal data analysis tool. Easy to use and reliable DIA analysis platforms have since been launched, in particular the popular commercial software Spectronaut (Bruderer et al., 2015), the open-source OpenSWATH (Rost et al., 2014), EncyclopeDIA (Searle et al., 2018), Skyline (Egertson et al., 2015), the more recently developed DIA-NN (Demichev et al., 2020), DIA-Umpire that allows DirectDIA (Tsou et al., 2015), and more [see overview in Zhang et al. (2020)].

## Application of DIA for Brain Research

DIA has been applied successfully for the comparative analysis of hippocampal synaptosome preparations from two rodent and two primate species (Koopmans et al., 2018b). A time-of-flight mass spectrometer was used for the measurement. The spectral library contained about 3,600 proteins that were generated from the same samples and measured on the same mass spectrometer operated in DDA mode. Peptides that were unique to one species were excluded for data analysis, resulting in the full comparative quantification of about 2,000 proteins. When comparing mouse with human, there were 644 proteins with higher abundance in human and 663 proteins with higher abundance in mouse. Of particular note were the sodium channels, in which specific subunits had a strong differential expression profile between rodents and primates. As sodium channels are involved in the formation and propagation of action potentials, the differences in expression pattern may qualify them for the distinct physiology of human neurons (Testa-Silva et al., 2014). When considering the changes globally, the differentially expressed proteins were highly enriched for synaptic plasticity-related processes. A similar mouse synaptosome preparation has also been analyzed by another high-end mass spectrometer, the Orbitrap Fusion Lumos, using a similar LC gradient as the previous experiment. About 4,600 proteins were quantified by DIA from a spectral library of about 5,000 proteins (Koopmans et al., 2018a), demonstrating the increase in protein coverage from technological advancement.

DIA has also been used for the analysis of whole cell lysates that are more complex than isolated organelles. Two recent studies have reported protein changes in primary neuronal culture after knockdown of candidate genes of interest (Rosato et al., 2019) or overexpression of a microRNA (He et al., 2018) related to schizophrenia. Currently, there are >100 known independent genetic risk loci associated with schizophrenia (Schizophrenia Working Group of the Psychiatric Genomics, 2014), but their contribution to the disease is unclear. Rosato et al. (2019) examined the neuronal phenotypes by knocking down 41 risk genes each by shRNA, and showed that three of them, Tcf4, Tbr1, and Top3b, caused similar changes in synaptic development. DIA analysis of the neuronal cultures with the individual knockdown of these three genes revealed limited overlap in protein changes. Interestingly, when the effect of Tcf4, Tbr1, and Top3b knockdown of all 210 regulated proteins was taken together, protein-protein interaction enrichment analysis identified a set of proteins involved in neurotransmitter release, including the core proteins SNAP25, SNAP29, NAPB, STX7, and STXBP5. This strongly suggests that polygenic risk in schizophrenia may converge onto common cellular pathways. The mir-137 locus is another genetic risk factor for schizophrenia. He et al. (2018) overexpressed mir-137 in primary neuronal culture, and showed by DIA that the proteins that changed significantly were enriched for cell adhesion and cell development, including NFASC, NLGN, NRXN, and HAPLN. Together with the electrophysiological and morphological data, it was concluded that mir-137 regulates synaptic function by regulating synaptogenesis, synaptic ultrastructure, and synapse function.

A recent study has used DIA to quantify the dynamics of a thousand surface N-glycoproteins in primary cortical neurons at 4, 6, 8, 16, 18, and 20 days *in vitro* (van Oostrum et al., 2020). Most surface protein abundance changes occurred within the first week prior to the time window for synapse formation. Interestingly, many synaptic proteins were present on the surface at the time when synapse counts began to increase, and they reached peak abundance 2 days before the peak of synapse counts at 18 days *in vitro*. This suggests that many synaptic proteins are produced and trafficked to the membrane surface before synapses are formed and only later are these proteins organized into synaptic micro-domains by surface diffusion. In another experimental paradigm that induced synaptic scaling by activation or inhibition of neuronal activity, about 30% of the surface proteins showed significant changes whereas total proteome showed <10% significant changes. This also suggests an extensive dynamic reorganization of the neuronal membrane surface that is largely independent of global protein abundance change. Both these studies demonstrate the successful implementation of DIA in the neuroscience field.

DIA is capable of analyzing very complex samples, such as a brain tissue extract. Bruderer (Bruderer et al., 2017) optimized the DIA protocol with a Q-Exactive HF mass spectrometer to examine the developmental changes of about 6,000 proteins in mouse somatosensory cortex 1-barrel field at P9, P15, P30, and P54. During early development, synaptic transmission-related proteins displayed a strong increase in expression and leveled off thereafter. Proteins of the mitochondrial respiratory chain showed a smooth increase over the whole developmental period. The down regulated proteins belonged to functional groups involved in axonogenesis, RNA splicing, or UBL conjugation processes.

DIA has also found application in some specific research fields, including the analysis of neuropeptides in mammals (Saidi et al., 2019) and in invertebrate model systems (DeLaney and Li, 2019), and the reporting of increase in detection of G-protein coupled receptors when a targeted virtual library is applied on top of the original project-specific spectral library (Lou et al., 2020).

## Recent Advancement of DIA

DIA is rapidly improving and approaching proteome-wide levels of detection. Advancements are achieved through better acquisition protocols and innovative computational data analysis (Figure 1), in conjunction with increased MS sensitivity and scan rate.

An early form of DIA, the MS^E^, was first introduced in 2005 (Silva et al., 2005). It uses a wide band-pass filter for precursor selection, resulting in highly complex fragment ion spectra in MS^E^ workflows that is challenging for data analysis. More recently, the coupling of an ion-mobility device to the mass spectrometer offers the possibility to fragment precursor ions after ion-mobility separation, thereby reducing complexity. Furthermore, the ramping of collision energies according to ion mobility improved the fragmentation efficiency and hence peptide identification rates (Distler et al., 2016). MS^E^ has been applied to study the effect of hippocampal proteins with the goal of determining protein alteration associated with low-dose whole body ionizing radiation on the changes of the hippocampal proteome. Out of about 400 identified proteins, about 70 have shown significant alteration (Huang et al., 2016). Using a similar approach, more than 2,000 proteins have been identified from post-synaptic density (Distler et al., 2014).

Recent algorithmic developments led to predicted spectral libraries that can be used instead of empirical libraries, with a performance on par in some settings/situations. Furthermore, the combined use of the predicted and empirical spectral libraries seems to improve the search results. Deep-learning-based models (Zhou et al., 2017; Gabriels et al., 2019; Tran et al., 2019) with neural networks have been applied on large DDA datasets to learn features of peptide fragment ions and their chromatographic retention time. For example, Prosit (Gessulat et al., 2019) used the ProteomeTools resource that contains 21,764,501 high-quality spectra from 576,256 unique precursors belonging to the synthetic peptide library that together covers 19,749 of the 20,040 human protein coding genes. The training enables subsequent prediction of relative intensities of peptide fragments and retention time, from which an extensive and precise predicted spectral library can be generated. One caveat to use all possible tryptic peptides in a database is its huge number contributing to false discovery rate correction, while the majority of the peptides are not detected. To mitigate this problem a hybrid method has been developed. A subset of the biological samples is analyzed by DIA with a narrow precursor isolation window and searched with the predicted spectral library (Searle et al., 2020; Van Puyvelde et al., 2020). The resulting data forms an empirically corrected library for database search. As the DIA injections for the empirically corrected library uses the same acquisition parameters, chromatographic conditions, and sample matrix as quantitative single injection DIA experiments, it gives an improved peptide detection rate over searching a project-specific DDA library. Recently, the deep learning model for spectrum prediction has been extended to include the post-translation modifications of the peptides (Zeng et al., 2019). An alternative strategy is to construct a hybrid library from the combined project-specific library with the directDIA analysis (Muntel et al., 2019; Zhong et al., 2020). With a LC gradient of 4 h or longer in a single shot analysis, >10,000 proteins have been quantified with the hybrid library at 1% protein FDR (Muntel et al., 2019).

The dynamic post-translation modification is an important molecular event that may change the properties of the protein, such as its activity, stability, subcellular localization, and interactions with other proteins. DIA is applicable to quantify post-translational modifications at a high through-put manner, as exemplified by a recent study (Bekker-Jensen et al., 2020). The phosphoproteome of retinal pigment epithelium cells was analyzed from 200 μg of whole-cell tryptic digests and enriched by Ti-IMAC. A short run time with 15-min liquid chromatography gradients identified about 20,000 phosphopeptides by DDA. The number of phosphopeptides was substantially increased to about 30,000 using a project-specific spectral library.

In addition, there are other less explored but equally promising DIA methodologies. In Scanning SWATH, precursor ions are fragmented using a “scanning” isolation window of 5 m/z, which is continuously scanned with the first quadrupole rather than the conventional DIA that used the stepwise windowed acquisition. The workflow quantified 2000–3000 proteins in 5–10 min run-time and can measure around 180 samples/day (https://doi.org/10.1101/656793). In another study, DIA has been coupled to the “parallel accumulation followed by serial fragmentation” in a trapped ion mobility quadrupole-TOF mass spectrometer for diaPASEF analysis (https://doi.org/10.1101/656207). Using a 17-min gradient separation (50 samples per day), about 6,000 proteins were quantified per sample. Higher throughput is feasible with 5 min gradients (180 samples per day), but the protein identification is then decreased to about 2,800 proteins. When a high resolution-high mass accuracy MS is used, DIA can be measured at the MS1 level, for example using WiSIM (Koopmans et al., 2018a) and BoxCar (Meier et al., 2018). In particular, the BoxCar-library-based workflow was shown to identify 10,000 protein groups from the mouse cerebellum extract in a single shot 100 min MS run (Meier et al., 2018).

## Alternative Methodologies Applicable to Quantitative Proteomics

It is reported that DIA is superior to DDA in reproducibility, specificity, and accuracy of relative protein quantification (Barkovits et al., 2020). Nevertheless, the apparent deficit of DDA can be offset with innovations in data analysis. The IonStar software demonstrated the quantification of 7,000 proteins in 100 brain samples with no missing data (Shen et al., 2018). A recent study demonstrated that using a spectral library search in DDA, in a manner similar to a DIA data analysis strategy, led to substantial improvement of reproducibility in protein identification and quantitation with lower coefficient of variation and reduced missing values (Fernandez-Costa et al., 2020).

In cases where a spectral library may not be readily obtainable, for instance when only limited input samples are available, DDA could be a better analysis choice. The use of a mass spectrometer gas phase cleavable chemical crosslinker to examine protein-protein interactions has recently been successfully applied to elucidate a global protein interactome in the synapse; around 12,000 unique lysine crosslinks from 2,362 proteins were identified (Gonzalez-Lozano et al., 2020). Given the stochastic nature of intra- and inter-protein crosslinking events at various lysine sites, a single spectral library for their identification is not favorable and therefore DDA was used. In experiments where the identification of peptides is the main goal, DDA is generally the method of choice.

Recently, proteomics analysis at the single cell level has been demonstrated, where isobaric labeling of samples with TMT measured by DDA improved sensitivity by the stacking effect of pooling TMT-labeled samples, in addition to the increase in through-put from the simultaneous analysis of multiple samples (Dou et al., 2019; Tsai et al., 2020).

For peptides specific to a low abundant protein-isoform or post-translational modifications present in a highly complex background, such as total tissue lysate, DIA may not have enough sensitivity and specificity for their detection and quantitation. In these cases, a targeted DIA strategy, the parallel reaction monitoring assay (Rauniyar, 2015), that focuses on tens to hundreds of pre-defined peptides and provides several fold higher sensitivity than DIA, could be a better choice, but only for the selected proteins of interest.

Therefore, for quantitative proteomics to be optimal the selection of a method, and specifically the advantages and limitations for the analysis of the samples, needs consideration. Isobaric multiplexing with isobaric labeling is advantageous for quantitation of a small sample size that matches the number of the available tags, for example a maximum of eight samples for iTRAQ and 16 for TMT. DDA is the method for label-free quantitation and is especially useful for experimental designs with only a few samples or specific applications, such as crosslink experiments. A parallel reaction monitoring assay has the highest sensitivity and specificity, and can be adopted for high throughput workflow on selected peptides/proteins. DIA is applicable to general quantitative proteomics. Due to its low missing value and good reproducibility, DIA has good potential for large-scale quantitative proteomics.

## Conclusion and Future Outlook

When is DIA the method of choice, and how could it advance our knowledge of brain function and disorders? Previous studies demonstrated the general applicability of DIA for neuroscience studies, including the analysis of the synapse, primary neuronal culture, brain tissues, human stem cells derived neurons and glial cells, and the dynamics of neuronal membrane proteins. These studies characterized the proteomes from mostly large populations of organelles or cells. For example, the synapse proteome characterized by proteomics is an average of all synapses isolated from a brain region (Pandya et al., 2017; Koopmans et al., 2018b). However, the size, shape, and functioning of synapses changes with neuronal activity, implicating compositional adaptation of their proteomes (Mansilla et al., 2018; Kulik et al., 2019). Important questions that have remained unanswered relate to the extent of the molecular diversity of synapses and how changes in synapse protein composition connect to neurological and psychiatric disorders. As DIA can be used for the analysis of multiple samples while maintaining good reproducibility and a low number of missing values, it would be used for systematic interrogation of these organelles under various conditions. Similar strategies will be applied to the studies of specific cell types and their proteome changes.

Considering that DIA has high-throughput capability with deep proteome analysis and good sensitivity, it is an advantageous method to be integrated in a platform for large-scale biomarker studies. For example, cerebrospinal fluid and blood proteome may be measured in a clinical environment as a routine diagnostical screen to reveal brain disorders and their disease stages. The feasibility to perform such large-scale analysis was recently demonstrated on the analysis of nearly 200 cerebrospinal fluid samples from Alzheimer's disease patients and healthy controls. Each DIA experiment quantified approximately a thousand proteins from a few microliters of sample (Bader et al., 2020). In the future, standardization of DIA protocols should be developed and adopted by large communities for meaningful comparison and interpretation of data, and to minimize batch effects across different laboratories.

Neurological and psychiatric disorders are frequently heterogeneous in nature. Each may further exhibit typical spatio-temporal malfunctions in the brain. Often, large sample sizes are necessary to reveal the global and subtype-specific analysis of the diseases. DIA, with its high reproducibility and low missing values, and good coverage of the proteome, is the preferred choice for such analysis. To accommodate comparative analysis of high numbers of samples, multi-laboratory projects would benefit from DIA. To be successful, standardization of protocols and hardware used for data acquisition, and the unified downstream data analysis, should be firmly established.

## Author Contributions

KL wrote the manuscript. MG-L drew the figure and critically reviewed the manuscript. FK provided input on all aspects of proteomics/DIA. AS assisted the writing of the manuscript. All authors contributed to the article and approved the submitted version.

## Conflict of Interest

The authors declare that the research was conducted in the absence of any commercial or financial relationships that could be construed as a potential conflict of interest.
